# Validity of a Self-Administered Food-Frequency Questionnaire for Assessing Amino Acid Intake in Japan: Comparison With Intake From 4-Day Weighed Dietary Records and Plasma Levels

**DOI:** 10.2188/jea.JE20150044

**Published:** 2016-01-05

**Authors:** Motoki Iwasaki, Junko Ishihara, Ribeka Takachi, Hidemi Todoriki, Hiroshi Yamamoto, Hiroshi Miyano, Taiki Yamaji, Shoichiro Tsugane

**Affiliations:** 1 Division of Epidemiology, Research Center for Cancer Prevention and Screening, National Cancer Center, Tokyo, Japan; 1 国立がん研究センターがん予防・検診研究センター疫学研究部; 2 Division of Prevention, Research Center for Cancer Prevention and Screening, National Cancer Center, Tokyo, Japan; 2 国立がん研究センターがん予防・検診研究センター予防研究部; 3 Research Center for Cancer Prevention and Screening, National Cancer Center, Tokyo, Japan; 3 国立がん研究センターがん予防・検診研究センター; 4 Department of Nutrition Management, Sagami Women’s University, Sagamigahara, Japan; 4 相模女子大学栄養科学部; 5 Department of Community Preventive Medicine, Division of Social and Environmental Medicine, Niigata University Graduate School of Medical and Dental Sciences, Niigata, Japan; 5 新潟大学大学院医歯学総合研究科地域予防医学講座環境予防医学分野; 6 Department of Environmental and Preventive Medicine, University of the Ryukyus, Nakagami-gun, Japan; 6 琉球大学医学部医学科衛生学・公衆衛生学分野; 7 Institute for Innovation, Ajinomoto Co., Inc., Kawasaki, Japan; 7 味の素株式会社イノベーション研究所

**Keywords:** amino acids, food frequency questionnaire, plasma level, validity, アミノ酸, 食物摂取頻度調査票, 血漿濃度, 妥当性

## Abstract

**Background:**

Interest in the physiological roles of amino acids and their impact on health outcomes is substantial and growing. This interest has prompted assessment of the habitual intake of amino acids for use in epidemiologic studies and in clarifying the association between habitual intake and plasma levels of amino acids. Here, we investigated the validity of ranking individuals according to dietary amino acid intake as estimated using a food frequency questionnaire (FFQ) in comparison with intakes from dietary records (DRs) and plasma levels.

**Methods:**

A total of 139 men and women selected from examinees of the cancer screening program at the Research Center for Cancer Prevention and Screening, National Cancer Center, Japan, provided 4-day weighed DRs, a semi-quantitative FFQ, and plasma samples. Plasma levels of amino acids were measured using the UF-Amino Station system.

**Results:**

Spearman rank correlation coefficients of energy-adjusted intake of amino acids from the DR and FFQ ranged from 0.40 to 0.65 for men and from 0.35 to 0.46 for women. Correlation coefficients of energy-adjusted intake from the DR and plasma levels ranged from −0.40 to 0.25 for men and from −0.16 to 0.11 for women. Similarly, no significant positive correlation coefficients were observed between intake from the FFQ and plasma levels for either men or women.

**Conclusions:**

We confirmed that this FFQ has moderate validity in estimating amino acid intake when 4-day weighed DRs are used as a reference method, suggesting that it is suitable for ranking individuals living in urban areas in Japan by amino acid intake.

## Abstract (ja)

**【背景】:**

アミノ酸の生理学的役割や健康アウトカムへの影響に対する関心は大きく、また高まっている。そこで、疫学研究に適用可能な習慣的なアミノ酸摂取の評価と、習慣的な摂取量と血中濃度との関連を明らかにすることが求められている。今回は、食物摂取頻度調査票から推定した食事由来のアミノ酸摂取量により個人をランキングすることの妥当性を、食事記録調査による摂取量および血中濃度と比較することにより検討した。

**【方法】:**

対象者は、国立がん研究センターがん予防・検診研究センターのがん検診受診者のうち、4日間の食事記録調査、半定量食物摂取頻度調査票、血漿検体を提供した139人である。血漿中アミノ酸濃度は、UF-amino Station systemを用いて分析した。

**【結果】:**

食事記録調査と食物摂取頻度調査票から得られたエネルギー調整済みアミノ酸摂取量のスピアマンの順位相関係数は、男性が0.40から0.65、女性が0.35から0.46であった。食事記録調査のエネルギー調整済み摂取量と血漿中濃度の相関係数は、男性が-0.40から0.25、女性が-0.16から0.11であった。同様に男女とも、食物摂取頻度調査票による摂取量と血漿中濃度の間にも正の相関は観察されなかった。

**【結論】:**

今回の食物摂取頻度調査票は、アミノ酸摂取量の推定において、4日間の食事記録調査を比較基準とした場合に中程度の妥当性があることを確認した。これは、日本の都市部在住者をアミノ酸摂取量によってランキングすることに適していることを示唆している。

## INTRODUCTION

The physiological roles of amino acids and their impact on health outcomes continue to attract strong research interest. Leucine, for example, one of the branched-chain amino acids (BCAAs), plays a key role in regulating muscle protein synthesis via the mammalian target of rapamycin (mTOR) signaling pathway and acts as a strong insulin secretagogue.^1^ Other examples include the associations between compromised insulin action and altered metabolism of amino acids,^2^ in which higher plasma levels of BCAAs are associated with the presence of obesity and visceral fat accumulation.^3^^,^^4^ A metabolomics-based study found that higher levels of BCAAs and aromatic amino acids were significantly associated with an increased risk of the development of diabetes.^5^

These research trends motivated us to assess the habitual intake of amino acids for use in epidemiologic studies and to clarify associations between habitual intake and the plasma levels of amino acids. We previously developed a comprehensive database of the amino acid content of foods using substitution methods and evaluated the validity of a self-administered food frequency questionnaire (FFQ) in estimating dietary amino acid intake among two populations in a validation study for the Japan Public Health Center-based Prospective Study (JPHC study).^6^ However, since the FFQ was developed for and validated in rural residents, it is unclear whether it accurately estimates dietary amino acid intake among urban residents in Japan, considering the ostensibly wider variety of foods eaten by urban dwellers in Japan than rural ones (percent energy from cereals among the former was less than that among the latter^7^). Further, to our knowledge, no study has comprehensively compared plasma levels of amino acids with dietary intake.

Here, we investigated the validity of ranking individuals according to dietary amino acid intake as estimated using a self-administered FFQ in comparison with intakes from 4-day weighed dietary records (DR) and plasma levels. This cross-sectional study was conducted using data from a previous validation study conducted among examinees of the cancer screening program at the Research Center for Cancer Prevention and Screening, National Cancer Center, Japan.

## MATERIALS AND METHODS

### Study population

Study subjects were participants in a validation study of a semi-quantitative FFQ used for a case-control study of colorectal adenoma in Tokyo, Japan.^8^^,^^9^ Details of this study have been described previously.^10^ Briefly, participants were selected from examinees of the cancer screening program at the Research Center for Cancer Prevention and Screening, National Cancer Center, Tokyo, Japan, from January 2004 through July 2006, who met the following criteria: 1) age between 40 and 69 years; 2) residence in Tokyo or surrounding suburbs; and 3) no previous or present diagnosis of cancer, cardiovascular disease, or diabetes mellitus. Examinees were stratified by sex and age (40–49, 50–59, and 60–69 years) and randomly numbered within each stratum. Potential participants were invited by ascending numbering order until the target number of participants was reached for each stratum. Among the 896 candidates invited, 187 agreed to participate in the study (response rate: 20.9%). After excluding those who could not attend the study orientation, 144 men and women provided weighed DRs over 4 consecutive days, a self-administered semi-quantitative FFQ, serum and EDTA-2Na plasma samples, and a 24-hour urine sample between May 2007 and April 2008. To avoid the metabolism of nonessential amino acids due to metabolic enzymes from blood cells, blood samples must be cooled to 0°C immediately after collection,^11^ so blood samples in the present study were left on ice and centrifuged within 30 minutes after collection. Aliquots of EDTA-2Na plasma were then prepared and stored frozen at −80°C within several hours of collection. The study was approved by the Institutional Review Board of the National Cancer Center, Tokyo, Japan. All participants provided written informed consent for participation at the study orientation.

### Dietary assessment

The 4-day weighed DR included 3 continuous weekdays and 1 weekend day. Food portions were measured by each participant during meal preparation using supplied digital scales and measuring spoons and cups. For foods purchased or consumed outside the home, the participants were instructed to record the approximate quantity of all foods in the meal and/or the names of the product and company. Trained dietitians checked the record with the participants and coded the foods and weights. Stores and restaurants were asked about the recipes of certain meals eaten outside the home.

The FFQ used for the present study was developed by modification of the FFQ used for the JPHC study, which was originally developed for and validated in rural residents. The food list was reviewed by experts in dietary assessment in epidemiological studies and modified by the exclusion of foods consumed in specific areas or at specific times and by the addition of foods consumed throughout the year in urban areas. The modified FFQ was validated in middle-aged urban participants undergoing cancer screening.^10^ The FFQ consisted of 138 food and beverage items with nine frequency categories and standard portions/units and asked about the usual consumption of listed foods during the previous year. Frequency response choices for food items were less than once per month, 1–3 times per month, 1–2 times per week, 3–4 times per week, 5–6 times per week, once per day, 2–3 times per day, 4–6 times per day, and 7 or more times per day. Standard portion sizes were specified for each food item in “amount” choices of small (50% smaller than standard), medium (standard) and large (50% larger). Daily food intake was calculated by multiplying frequency by standard portion and relative size for each food item.

Intakes of energy and protein were calculated using the Standardized Tables of Food Composition in Japan, 5th revised and enlarged edition, which include 1878 food items.^12^ Because the database for amino acids, published as the ‘Standardized Tables of Food Composition in Japan, Amino Acid Composition of Foods 2010’, covered only 18% of these items (337 food items),^13^ we updated a previously developed comprehensive database of the amino acid content of foods using substitution methods.^6^ Daily nutrient intakes for each individual were calculated by summing the product of the intake of each food multiplied by the nutrient content of that food. Because a database for dietary supplements was not available, intake from dietary supplements was not included in calculations for either the DR or FFQ.

### Laboratory analysis

A 75-µL portion of plasma sample was added to 75 µL of internal standard (isotope-labeled amino acid) solution and 150 µL of acetonitrile. The solution was mixed in a vortex mixer and then centrifuged at 15 000 rpm for 10 min at 20°C.

Amino acid analysis was performed using a UF-Amino Station (Shimadzu Corporation, Kyoto, Japan), equipped with a pump, autosampler, column oven, and mass spectrometer. Separation was performed on a Shim-pack UF-Amino column (100 mm × 2.1 mm; 2 µm) (Shimadzu Corporation, Kyoto, Japan) and eluted with 25 mM ammonium formate in water (pH 6.0) and acetonitrile (Wako Pure Chemical Industries, Osaka, Japan). 3-aminopyridyl-N-hydroxysuccinimidyl carbamate (20 mM) in acetonitrile and 200 mM borate buffer (pH 9.2) were prepared for derivatization of amino acids.

The UF-Amino Station has four features: 1) ultra-high speed analysis that separates 35 amino acids in 9 minutes; 2) automatic precolumn derivatization; 3) a dedicated workstation, AmiNavi^®^; and 4) superior selectivity from matrices. In this study, the following 18 compounds were measured: alanine, arginine, aspartic acid, cystine, glutamic acid, glycine, histidine, isoleucine, leucine, lysine, methionine, phenylalanine, proline, serine, threonine, tryptophan, tyrosine, and valine. All assays were performed at the Frontier Research Labs., Institute for Innovation, Ajinomoto Co., Inc. (Kawasaki, Japan) Blind duplicate plasma samples from two subjects were included on three different days as quality controls. All intra-assay coefficients of variation (CVs) were 6% or lower except for cystine (6.2%) and aspartic acid (14.2% and 17.8%), and all inter-CVs were 6% or lower except for cystine (10.1% and 6.2%), aspartic acid (16.9% and 14.9%), and glutamic acid (11.4%).

### Statistical analysis

We excluded subjects whose 4-day weighed DRs were not available and who reported extremely low or high total energy intake in the FFQ (<800 or ≥4000 kcal), leaving 139 men and women for inclusion in the present analyses.

Measurement values below the limit of quantitation (LOQ) were assigned half the value of the LOQ. All amino acids were over the LOQ in all samples measured except for aspartic acid in one sample. Dietary amino acid intake according to the DR and FFQ was adjusted for total energy intake using the residual method. All analyses were performed by sex. First, we calculated mean intakes of amino acids according to both the DR and FFQ and mean plasma levels of amino acids. We then calculated Spearman rank correlation coefficients and 95% confidence intervals between intakes according to the DR and FFQ for crude and energy-adjusted values. Correlation coefficients were deattenuated using the following formula: $\sqrt{\left(1+\frac{\lambda }{\text{n}}\right)}$, where n is the number of DRs, and *λ* is the ratio of within- to between-individual variance based on the 4-day DRs. Similarly, we also calculated Spearman rank correlation coefficients and 95% confidence intervals between plasma level and intakes according to the DR and FFQ for crude and energy-adjusted values. In addition, to validate the categorization of subjects, we computed the number of subjects classified into the same, adjacent, and extreme categories by joint classification by quintile and provided weighted kappa coefficients. For comparison of intakes between DRs and FFQ, limits of agreement (LOAs) were calculated in order to assess agreement between the two methods. Namely, the difference between the FFQ and DR (FFQ-DR) and the average of the FFQ and DR ([FFQ + DR]/2) was calculated for each subject. The LOAs defined the boundaries within which 95% of all the differences between methods were expected to fall and were calculated as mean difference ± *t*_(n−1,0.025)_ (standard deviation of differences). Any dependency between the two methods was tested by fitting the regression line of differences. Because all dietary intakes were log-transformed for this analysis, the antilog of the mean difference and LOAs was taken, providing a ratio of FFQ/DR for the data. The ratios were multiplied by 100 and are therefore expressed as percentages.^14^ All statistical analyses were performed using SAS software version 9.3 (SAS Institute, Inc., Cary, NC, USA).

## RESULTS

Characteristics of study subjects are presented in Table 1. Data from 68 men and 71 women were analyzed, with mean ages of 58.8 and 58.4 years, respectively. Mean energy intake tended to be higher for men (2272 kcal) than women (1834 kcal).

**Table 1.** Characteristics of study subjects

	Men (*n* = 68)			Women (*n* = 71)		
	
	Mean	Standard deviation	Median	Mean	Standard deviation	Median
Age, years	58.8	7.3	59.0	58.4	7.4	59.0
Body mass index, kg/m^2^	23.3	2.5	23.3	21.5	2.5	21.4
Energy, kcal^a^	2272	419	2209	1834	300	1806
Protein, g^a^	90.4	15.8	90.0	75.5	13.6	74.2
Fat, g^a^	65.2	14.3	63.8	57.6	16.4	54.8
Carbohydrates, g^a^	298.3	70.4	285.3	243.4	46.2	243.9

^a^Estimated using dietary records.

Mean intake of amino acids, assessed with the DR and FFQ, and mean level of plasma amino acids are shown in Table 2. Overall, mean intake of amino acids tended to be higher using the DR than using the FFQ. Among the 18 amino acids, mean intake was highest for glutamic acid and lowest for tryptophan for both men and women, regardless of dietary assessment method. Meanwhile, mean plasma level was highest for alanine and lowest for aspartic acid for both men and women.

**Table 2.** Amino acid intake assessed with dietary records and food frequency questionnaire, and plasma amino acid level

	Dietary records			Food frequency questionnaire			Plasma level		
		
	Mean (mg/day)	Standard deviation	Median (mg/day)	Mean (mg/day)	Standard deviation	Median (mg/day)	Mean (µmol/L)	Standard deviation	Median (µmol/L)
Men (*n* = 68)									
Isoleucine	3765	675	3744	3180	1446	2894	71.3	18.9	68.2
Leucine	6700	1182	6656	5758	2557	5323	133.2	26.0	131.5
Lysine	5744	1187	5796	4761	2361	4162	197.3	34.5	195.0
Methionine	1990	398	2018	1677	793	1489	27.4	7.1	26.9
Phenylalanine	3868	663	3888	3313	1423	3108	62.1	7.6	61.4
Threonine	3455	646	3432	2875	1313	2620	124.2	24.5	121.4
Tryptophan	1042	184	1033	892	392	826	58.4	9.0	57.8
Valine	4427	791	4437	3781	1689	3460	244.3	38.1	244.7
Histidine	2958	659	2929	2468	1210	2172	81.9	7.9	82.1
Arginine	5260	1045	5224	4259	1831	3909	91.2	15.1	90.7
Cystine	1295	224	1302	1095	454	1021	23.8	6.8	23.2
Tyrosine	2979	536	2974	2596	1171	2392	66.2	12.6	64.9
Alanine	4373	882	4374	3529	1577	3244	373.9	74.2	362.8
Aspartic acid	8192	1624	8220	6710	3005	6061	3.1	1.0	2.9
Glutamic acid	15 872	2458	15 844	13 325	5464	12 108	49.2	19.1	43.6
Glycine	3904	809	3833	3052	1336	2822	202.1	38.7	195.7
Proline	4870	822	4860	4307	1860	4022	161.7	44.5	158.5
Serine	3964	695	3974	3358	1483	3086	109.5	18.7	107.6
Women (*n* = 71)									
Isoleucine	3151	599	3144	2818	1020	2625	61.8	20.3	57.2
Leucine	5601	1051	5618	5093	1822	4698	112.6	28.2	105.2
Lysine	4789	1038	4802	4219	1608	3949	182.8	32.8	186.6
Methionine	1649	342	1644	1476	533	1379	24.9	5.6	23.9
Phenylalanine	3219	573	3188	2934	1022	2775	59.7	9.7	58.2
Threonine	2886	566	2878	2551	909	2410	123.1	31.9	115.2
Tryptophan	869	157	867	793	276	742	53.9	8.6	53.4
Valine	3703	697	3645	3348	1211	3132	215.1	46.3	204.9
Histidine	2387	562	2370	2188	820	2012	80.2	10.2	79.1
Arginine	4324	836	4329	3762	1272	3741	91.0	23.5	88.7
Cystine	1071	180	1054	971	318	946	19.4	5.7	17.8
Tyrosine	2485	465	2460	2294	840	2151	66.1	14.3	61.6
Alanine	3627	755	3585	3132	1068	3017	349.4	68.0	347.2
Aspartic acid	6801	1327	6881	5951	2094	5721	2.8	1.1	2.6
Glutamic acid	13 375	2251	13 030	12 169	4337	11 634	40.0	17.9	37.5
Glycine	3217	741	3095	2707	923	2602	242.3	67.7	225.3
Proline	4172	824	4072	3953	1520	3682	137.2	41.8	127.2
Serine	3338	599	3306	2988	1053	2812	120.4	25.0	117.6

Spearman rank correlation coefficients of amino acid intake from the DR and FFQ are presented in Table 3. Correlation coefficients based on crude intake ranged from 0.28 to 0.45 for men and from 0.49 to 0.63 for women. Correlation coefficients based on energy-adjusted intake tended to be higher than those based on crude intake for men, ranging from 0.40 to 0.65, but tended to be lower in women, ranging from 0.35 to 0.46. In addition, correlation coefficients after deattenuation tended to be higher than those based on energy-adjusted intake. Overall, significant correlation coefficients were observed regardless of sex or method used to estimate amino acids.

**Table 3.** Spearman rank correlation coefficients of amino acid intake from dietary records and a food frequency questionnaire

	Men (*n* = 68)					Women (*n* = 71)				
	
	Crude intake		Energy-adjusted intake			Crude intake		Energy-adjusted intake		
			
	CC	95% confidence interval	CC	95% confidence interval	De- attenuated CC	CC	95% confidence interval	CC	95% confidence interval	De- attenuated CC
Protein	0.35	(0.12, 0.54)	0.54	(0.35, 0.69)	0.67	0.55	(0.36, 0.69)	0.39	(0.18, 0.57)	0.47
Isoleucine	0.38	(0.16, 0.57)	0.59	(0.41, 0.73)	0.75	0.58	(0.40, 0.71)	0.43	(0.22, 0.60)	0.52
Leucine	0.38	(0.15, 0.56)	0.58	(0.39, 0.72)	0.73	0.58	(0.40, 0.71)	0.37	(0.15, 0.56)	0.46
Lysine	0.37	(0.14, 0.56)	0.57	(0.38, 0.71)	0.73	0.57	(0.39, 0.71)	0.42	(0.20, 0.59)	0.52
Methionine	0.36	(0.13, 0.55)	0.54	(0.34, 0.69)	0.69	0.55	(0.36, 0.69)	0.39	(0.18, 0.58)	0.49
Phenylalanine	0.40	(0.18, 0.58)	0.59	(0.41, 0.72)	0.73	0.57	(0.39, 0.71)	0.42	(0.21, 0.60)	0.52
Threonine	0.36	(0.13, 0.55)	0.56	(0.37, 0.70)	0.70	0.56	(0.38, 0.70)	0.43	(0.21, 0.60)	0.52
Tryptophan	0.40	(0.18, 0.58)	0.61	(0.43, 0.74)	0.74	0.60	(0.43, 0.73)	0.44	(0.23, 0.61)	0.54
Valine	0.39	(0.17, 0.57)	0.59	(0.41, 0.73)	0.73	0.58	(0.40, 0.72)	0.42	(0.20, 0.59)	0.50
Histidine	0.32	(0.09, 0.52)	0.40	(0.18, 0.59)	0.60	0.63	(0.47, 0.76)	0.46	(0.26, 0.63)	0.61
Arginine	0.44	(0.22, 0.61)	0.57	(0.39, 0.71)	0.69	0.49	(0.29, 0.65)	0.38	(0.17, 0.57)	0.48
Cystine	0.45	(0.23, 0.62)	0.51	(0.31, 0.67)	0.60	0.51	(0.31, 0.66)	0.37	(0.15, 0.56)	0.45
Tyrosine	0.40	(0.17, 0.58)	0.58	(0.39, 0.72)	0.72	0.62	(0.45, 0.74)	0.46	(0.25, 0.63)	0.57
Alanine	0.39	(0.17, 0.58)	0.61	(0.43, 0.74)	0.75	0.53	(0.33, 0.68)	0.39	(0.17, 0.57)	0.47
Aspartic acid	0.38	(0.16, 0.57)	0.65	(0.49, 0.77)	0.78	0.54	(0.35, 0.69)	0.43	(0.22, 0.60)	0.52
Glutamic acid	0.30	(0.07, 0.50)	0.56	(0.37, 0.71)	0.72	0.51	(0.31, 0.66)	0.35	(0.13, 0.54)	0.43
Glycine	0.38	(0.16, 0.57)	0.48	(0.27, 0.64)	0.61	0.53	(0.33, 0.68)	0.35	(0.13, 0.54)	0.42
Proline	0.28	(0.05, 0.49)	0.57	(0.39, 0.71)	0.75	0.55	(0.36, 0.69)	0.35	(0.13, 0.54)	0.42
Serine	0.37	(0.15, 0.56)	0.55	(0.36, 0.70)	0.67	0.53	(0.34, 0.68)	0.36	(0.13, 0.54)	0.43

CC, correlation coefficient.

Spearman rank correlation coefficients of amino acid intake from the DR and plasma amino acid levels are shown in Table 4. Overall, no significant correlation coefficients were observed regardless of sex or estimation method, albeit with several exceptions. Lysine for men showed weak positive correlation coefficients using both crude and energy-adjusted intake. Inverse correlation coefficients were observed for histidine using both crude and energy-adjusted intake in men, for glycine using energy-adjusted intake in men and crude intake in women, and for proline using crude intake in men. These inverse correlation coefficients did not substantially change when subjects with non-fasting samples (less than 8 hours) were excluded (data not shown). In contrast, exclusion resulted in loss of the significant positive correlation coefficients for both crude and energy-adjusted lysine intake among men. In addition, partial correlation coefficients after adjustment for urinary creatinine level showed similar results, since this level reflects whole-body muscle volume, which may affect plasma levels for some essential amino acids (data not shown).

**Table 4.** Spearman rank correlation coefficients of amino acid intake from dietary records and plasma amino acid level

	Men (*n* = 68)				Women (*n* = 71)			
	
	Crude intake		Energy-adjusted intake		Crude intake		Energy-adjusted intake	
			
	CC	95% confidence interval	CC	95% confidence interval	CC	95% confidence interval	CC	95% confidence interval
Isoleucine	−0.15	(−0.37, 0.09)	−0.12	(−0.35, 0.13)	−0.01	(−0.24, 0.22)	−0.04	(−0.27, 0.20)
Leucine	−0.01	(−0.25, 0.23)	−0.02	(−0.26, 0.22)	0.002	(−0.23, 0.24)	−0.08	(−0.31, 0.16)
Lysine	0.25	(0.01, 0.46)	0.25	(0.01, 0.46)	−0.01	(−0.24, 0.22)	0.08	(−0.16, 0.31)
Methionine	0.13	(−0.11, 0.36)	−0.06	(−0.29, 0.18)	0.04	(−0.19, 0.27)	0.11	(−0.13, 0.33)
Phenylalanine	0.02	(−0.21, 0.26)	−0.02	(−0.26, 0.22)	0.04	(−0.19, 0.27)	−0.01	(−0.24, 0.22)
Threonine	−0.002	(−0.24, 0.24)	−0.12	(−0.35, 0.12)	0.02	(−0.22, 0.25)	0.06	(−0.17, 0.29)
Tryptophan	−0.05	(−0.29, 0.19)	−0.10	(−0.33, 0.14)	0.01	(−0.23, 0.24)	0.05	(−0.19, 0.28)
Valine	−0.03	(−0.27, 0.21)	0.05	(−0.19, 0.29)	0.04	(−0.20, 0.27)	−0.09	(−0.32, 0.14)
Histidine	−0.27	(−0.47, −0.03)	−0.40	(−0.58, −0.18)	0.17	(−0.07, 0.38)	0.11	(−0.13, 0.33)
Arginine	0.07	(−0.17, 0.31)	0.05	(−0.19, 0.28)	−0.02	(−0.25, 0.21)	0.10	(−0.13, 0.33)
Cystine	0.16	(−0.08, 0.39)	0.13	(−0.11, 0.36)	0.01	(−0.22, 0.25)	−0.07	(−0.30, 0.17)
Tyrosine	−0.07	(−0.31, 0.17)	−0.17	(−0.40, 0.07)	−0.05	(−0.28, 0.18)	0.00	(−0.23, 0.23)
Alanine	−0.23	(−0.45, 0.01)	−0.23	(−0.44, 0.01)	−0.02	(−0.25, 0.21)	0.02	(−0.22, 0.25)
Aspartic acid	0.14	(−0.10, 0.37)	0.10	(−0.14, 0.33)	0.18	(−0.06, 0.40)	0.03	(−0.20, 0.26)
Glutamic acid	0.08	(−0.16, 0.32)	−0.01	(−0.25, 0.23)	−0.03	(−0.26, 0.21)	−0.05	(−0.28, 0.18)
Glycine	−0.18	(−0.40, 0.06)	−0.25	(−0.46, −0.01)	−0.25	(−0.46, −0.02)	−0.16	(−0.38, 0.07)
Proline	−0.26	(−0.47, −0.02)	−0.15	(−0.37, 0.09)	−0.13	(−0.35, 0.11)	−0.03	(−0.26, 0.20)
Serine	−0.07	(−0.31, 0.17)	0.01	(−0.23, 0.24)	−0.11	(−0.33, 0.13)	0.05	(−0.18, 0.28)

CC, correlation coefficient.

Spearman rank correlation coefficients of amino acid intake from the FFQ and plasma amino acid levels are shown in Table 5. No significant positive correlation coefficients were observed, regardless of sex or estimation method, whereas significant inverse correlation coefficients were found for isoleucine using crude intake in men, cysteine using energy-adjusted intake in men, alanine using both crude and energy-adjusted intake in men, and glycine using crude and energy-adjusted intake in women. These results did not substantially change when subjects with non-fasting samples (less than 8 hours) were excluded (data not shown). Moreover, these results did not substantially change after further adjustment for urinary creatinine level (data not shown).

**Table 5.** Spearman rank correlation coefficients of amino acid intake from a food frequency questionnaire and plasma amino acid level

	Men (*n* = 68)				Women (*n* = 71)			
	
	Crude intake		Energy-adjusted intake		Crude intake		Energy-adjusted intake	
			
	CC	95% confidence interval	CC	95% confidence interval	CC	95% confidence interval	CC	95% confidence interval
Isoleucine	−0.32	(−0.52, −0.09)	−0.12	(−0.35, 0.12)	−0.14	(−0.36, 0.09)	−0.15	(−0.37, 0.08)
Leucine	−0.21	(−0.43, 0.02)	−0.07	(−0.31, 0.17)	−0.12	(−0.34, 0.12)	−0.13	(−0.36, 0.10)
Lysine	−0.06	(−0.29, 0.18)	0.11	(−0.13, 0.34)	0.02	(−0.21, 0.25)	0.08	(−0.16, 0.30)
Methionine	−0.09	(−0.32, 0.15)	−0.16	(−0.38, 0.08)	−0.02	(−0.26, 0.21)	0.00	(−0.23, 0.24)
Phenylalanine	−0.11	(−0.34, 0.13)	−0.12	(−0.35, 0.12)	−0.05	(−0.28, 0.19)	−0.10	(−0.33, 0.13)
Threonine	−0.18	(−0.40, 0.06)	−0.17	(−0.39, 0.07)	0.10	(−0.13, 0.33)	0.13	(−0.10, 0.36)
Tryptophan	−0.10	(−0.33, 0.14)	0.00	(−0.24, 0.24)	0.01	(−0.23, 0.24)	0.03	(−0.20, 0.27)
Valine	−0.16	(−0.38, 0.09)	0.05	(−0.19, 0.28)	−0.05	(−0.28, 0.18)	−0.10	(−0.32, 0.14)
Histidine	−0.02	(−0.26, 0.22)	−0.09	(−0.32, 0.15)	0.10	(−0.13, 0.33)	0.07	(−0.17, 0.30)
Arginine	−0.08	(−0.31, 0.16)	0.08	(−0.16, 0.31)	−0.01	(−0.24, 0.22)	0.03	(−0.20, 0.26)
Cystine	−0.17	(−0.40, 0.07)	−0.27	(−0.48, −0.03)	−0.14	(−0.36, 0.09)	−0.16	(−0.38, 0.08)
Tyrosine	−0.01	(−0.25, 0.23)	−0.12	(−0.35, 0.12)	0.05	(−0.19, 0.28)	0.08	(−0.16, 0.31)
Alanine	−0.27	(−0.48, −0.04)	−0.25	(−0.46, −0.01)	0.01	(−0.23, 0.24)	0.02	(−0.21, 0.25)
Aspartic acid	−0.06	(−0.29, 0.18)	−0.06	(−0.29, 0.18)	0.16	(−0.08, 0.38)	0.15	(−0.08, 0.37)
Glutamic acid	0.06	(−0.18, 0.29)	−0.12	(−0.35, 0.12)	0.02	(−0.22, 0.25)	0.05	(−0.18, 0.28)
Glycine	−0.19	(−0.41, 0.05)	−0.14	(−0.37, 0.10)	−0.28	(−0.48, −0.05)	−0.25	(−0.46, −0.02)
Proline	−0.23	(−0.45, 0.00)	0.03	(−0.21, 0.26)	−0.08	(−0.31, 0.16)	0.00	(−0.23, 0.23)
Serine	−0.09	(−0.32, 0.15)	−0.05	(−0.28, 0.19)	−0.05	(−0.28, 0.19)	0.03	(−0.21, 0.26)

CC, correlation coefficient.

Table 6 and Table 7 compare amino acid intake from the DR and FFQ and amino acid levels in plasma based on joint classification by quintile. Percentages of the same and adjacent category ranged from 71% to 82% for men and from 63% to 76% for women, while percentages of the extreme category were less than 3% for both men and women on comparison of amino acid intake from the DR and FFQ. Weighted kappa values showed moderate agreement (0.40–0.60), except for histidine in men and proline in women. In contrast, percentages of the same and adjacent category were less than 62% for both men and women and percentages of the extreme category ranged from 3% to 16% on comparison of dietary intake and plasma levels of amino acids. Weighted kappa values showed poor agreement (<0.20), except for lysine from dietary records and plasma levels in men.

**Table 6.** Comparison of amino acid intake from DRs and a FFQ based on joint classification by quintile and agreement between the DR and FFQ for amino acid intake

	Same category (%)	Same and adjacent category (%)	Extreme category (%)	Weighted Kappa	Mean (%)^a^	95% confidence interval	95% limit of agreement^b^	Regression coefficient^c^	*P* value
Men									
Isoleucine	43	78	0	0.62	79	(76, 84)	(53, 120)	0.69	<0.05
Leucine	34	76	0	0.57	81	(77, 85)	(54, 121)	0.71	<0.05
Lysine	34	76	1	0.52	77	(73, 82)	(47, 127)	0.59	<0.05
Methionine	32	75	0	0.55	79	(75, 84)	(50, 125)	0.58	<0.05
Phenylalanine	38	78	1	0.54	81	(78, 85)	(56, 117)	0.71	<0.05
Threonine	32	79	1	0.52	78	(74, 83)	(51, 120)	0.63	<0.05
Tryptophan	44	76	1	0.57	81	(77, 85)	(56, 118)	0.67	<0.05
Valine	46	78	0	0.59	81	(77, 85)	(54, 119)	0.67	<0.05
Histidine	29	71	3	0.37	78	(73, 84)	(46, 135)	0.53	<0.05
Arginine	31	78	1	0.54	77	(73, 81)	(52, 113)	0.47	<0.05
Cystine	37	75	1	0.50	81	(77, 84)	(58, 112)	0.71	<0.05
Tyrosine	35	79	1	0.57	82	(78, 86)	(55, 123)	0.69	<0.05
Alanine	38	82	1	0.60	76	(73, 80)	(51, 115)	0.52	<0.05
Aspartic acid	41	79	1	0.62	78	(74, 81)	(53, 114)	0.48	<0.05
Glutamic acid	35	75	0	0.56	80	(76, 83)	(56, 115)	0.81	<0.05
Glycine	32	74	3	0.47	74	(70, 78)	(47, 117)	0.52	<0.05
Proline	35	75	1	0.51	84	(79, 88)	(54, 128)	0.73	<0.05
Serine	37	75	0	0.54	80	(76, 84)	(55, 117)	0.70	<0.05
Women									
Isoleucine	27	72	0	0.51	84	(81, 87)	(62, 115)	0.25	0.09
Leucine	31	65	0	0.47	86	(83, 89)	(63, 117)	0.26	0.09
Lysine	27	73	0	0.50	83	(79, 87)	(57, 121)	0.21	0.17
Methionine	31	65	0	0.47	85	(81, 89)	(59, 122)	0.21	0.21
Phenylalanine	30	75	0	0.53	86	(83, 89)	(65, 114)	0.31	<0.05
Threonine	28	69	0	0.51	83	(80, 87)	(61, 114)	0.22	0.15
Tryptophan	31	75	1	0.51	86	(83, 89)	(65, 114)	0.30	<0.05
Valine	31	72	0	0.50	85	(82, 88)	(63, 115)	0.27	0.08
Histidine	38	76	0	0.62	86	(82, 91)	(58, 129)	0.15	0.29
Arginine	30	69	1	0.45	82	(79, 85)	(61, 112)	0.27	0.09
Cystine	27	73	3	0.45	86	(83, 88)	(66, 111)	0.42	<0.05
Tyrosine	32	75	0	0.58	87	(84, 90)	(64, 118)	0.27	0.06
Alanine	25	72	1	0.48	82	(79, 85)	(59, 114)	0.20	0.23
Aspartic acid	35	73	0	0.52	83	(80, 86)	(61, 112)	0.27	0.07
Glutamic acid	31	69	1	0.44	85	(83, 88)	(65, 112)	0.27	0.08
Glycine	31	66	3	0.48	80	(77, 84)	(56, 115)	0.15	0.39
Proline	28	63	1	0.39	89	(86, 92)	(65, 122)	0.17	0.28
Serine	32	72	1	0.48	84	(82, 87)	(64, 112)	0.32	<0.05

DR, dietary record; FFQ, food frequency questionnaire.

^a^Exp (mean [FFQ-DR]) as a multiple of the DR (all dietary intake data were log-transformed).

^b^Mean difference ± *t*_(n−1,0.025)_ (standard deviation of differences).

^c^Slope of mean of methods regressed on difference between methods.

**Table 7.** Comparison of plasma amino acids with those from dietary records and a food frequency questionnaire based on joint classification by quintile

	Dietary record vs plasma level								Food frequency questionnaire vs plasma level							
	
	Men				Women				Men				Women			
			
	Same category (%)	Same and adjacent category (%)	Extreme category (%)	Weighted Kappa	Same category (%)	Same and adjacent category (%)	Extreme category (%)	Weighted Kappa	Same category (%)	Same and adjacent category (%)	Extreme category (%)	Weighted Kappa	Same category (%)	Same and adjacent category (%)	Extreme category (%)	Weighted Kappa
Isoleucine	12	49	10	−0.08	23	56	10	−0.05	19	46	12	−0.15	18	48	11	−0.15
Leucine	25	51	7	0.01	24	52	11	−0.12	21	53	9	−0.04	17	48	10	−0.13
Lysine	19	57	3	0.24	18	62	10	0.07	15	50	9	0.04	21	55	8	0.08
Methionine	19	46	7	−0.05	18	59	8	0.09	18	47	9	−0.16	20	55	10	−0.05
Phenylalanine	18	56	7	0.02	28	49	8	0.01	24	53	10	−0.07	17	42	8	−0.09
Threonine	25	46	7	−0.11	25	54	8	0.05	15	43	6	−0.10	23	54	6	0.12
Tryptophan	18	50	12	−0.12	25	55	8	0.08	16	60	9	0.04	20	54	8	0.07
Valine	21	57	7	0.10	18	55	10	−0.07	19	54	4	0.10	18	35	8	−0.10
Histidine	18	40	15	−0.34	25	51	6	0.08	21	50	10	−0.03	15	55	8	0.07
Arginine	19	60	7	0.06	23	54	7	0.08	22	57	10	0.03	17	48	7	0.03
Cystine	16	62	7	0.12	28	46	7	−0.08	13	44	15	−0.28	20	45	10	−0.14
Tyrosine	15	44	16	−0.19	18	52	11	−0.10	21	51	15	−0.17	24	52	7	0.08
Alanine	12	43	9	−0.27	20	52	8	−0.03	15	44	13	−0.29	23	51	10	−0.02
Aspartic acid	32	56	7	0.17	17	54	6	0.04	24	59	10	−0.03	31	61	7	0.17
Glutamic acid	21	54	9	0.03	15	42	6	−0.06	22	49	9	−0.07	23	58	11	0.01
Glycine	13	44	13	−0.35	15	42	11	−0.16	19	50	12	−0.13	17	44	13	−0.26
Proline	13	38	10	−0.15	20	58	10	0.04	19	51	6	0.00	18	55	10	0.00
Serine	19	53	7	−0.05	21	62	8	0.12	26	50	7	−0.07	11	51	7	0.03

Agreement between the two methods is assessed in Table 6. The FFQ estimate ranged from 74% to 84% of the DR estimate for men and from 80% to 89% for women. The LOAs indicate that the FFQ may underestimate amino acid intake by 42% to 54% and overestimate it by 12% to 35% in men, and underestimate by 34% to 44% and overestimate by 11% to 29% in women. Regression coefficients were positive, particularly with regard to all values that were statistically significant in men. This indicates that agreement became worse with increasing intake.

## DISCUSSION

In this study, we found moderate correlation coefficients for amino acid intake between estimations using DR and FFQ. In contrast, we saw no positive correlation coefficient between intakes from the DR and FFQ and plasma amino acid levels.

Our previous study evaluated the validity of an FFQ in estimating dietary amino acid intake among two populations in the validation study of the JPHC study, which used 28-day weighed DRs as a reference standard.^6^ Spearman rank correlation coefficients of energy-adjusted intake from 18 amino acids ranged from 0.29 to 0.38 for men and from 0.14 to 0.30 for women in the first group from the population the FFQ was developed for, and from 0.29 to 0.50 for men and from 0.26 to 0.44 for women in a second group from a separate population used to confirm external validity. These findings are somewhat lower than our present findings. This difference might be partly explained by differences in the validity of protein intake, with Spearman rank correlation coefficients of energy-adjusted protein intake in the JPHC validation study of 0.33 for men and 0.22 for women in the first group and 0.22 for men and 0.16 for women in the second group^6^ versus 0.54 for men and 0.39 for women in the present study.

These differences in the validity of protein intake between the two studies might be explained by several methodological differences. First, study subjects were residents of rural areas in the JPHC validation study, while subjects were residents of urban areas in the present study. In particular, participants in the present study were randomly selected and recruited from examinees of a cancer screening program, and the response rate was low, which may have resulted in a higher proportion of health-conscious subjects than the actual population. Therefore, the possibility that the validity of the FFQ was overestimated cannot be ruled out.

Second, the FFQ used in the present study, which was initially developed for the JPHC study, was modified for a middle-aged urban population. In particular, 10 foods mainly consumed in specific areas or at specific times were excluded (luncheon meats, vivipara [a univalve shell living in freshwater, such as in a paddy field], *qing-geng-cai* [bok choy], leaf mustard, bitter gourd, chard, loofah, mugwort, *yushi-tofu* [soft, boiled tofu], and Okinawa soba) and 11 foods consumed throughout the year in urban areas were added (beef, stir-fried; chicken, stir-fried; chicken, stewed; low-fat milk; Japanese amberjack; Welsh onion; eggplant; edible burdock; konnyaku foods [devil’s tongue]; *nama-age* [fried slices of drained tofu]; and jam, strawberry or marmalade).^10^ We calculated the cumulative percentage contributions of these foods to protein intake and found that the cumulative percentage of the 11 added foods was 7.3% for men and 6.8% for women, whereas that of the 10 excluded foods was 1.8% for men and 1.4% for women in the first group and 1% for both men and women in the second group. Thus, modification of the FFQ might improve the validity of protein intake.

Third, reference intakes were evaluated using 28-day weighed DRs in the validation study of the JPHC study but using 4-day weighed DRs in our present study. Our previous studies reported deattenuated Spearman rank correlation coefficients, which were corrected for the attenuating effect of random intra-individual error from the usual intake.^6^^,^^10^ Deattenuated correlation coefficients for protein intake (0.67 for men and 0.47 for women) were somewhat higher than energy-adjusted values (0.55 for men and 0.39 for women) among examinees of the cancer screening program at the Research Center for Cancer Prevention and Screening, National Cancer Center.^10^ In contrast, energy-adjusted and deattenuated correlation coefficients for protein intake in the validation study of the JPHC study were similar: energy-adjusted correlation coefficients were 0.33 for men and 0.22 for women in the first group and 0.22 for men and 0.16 for women in the second, while deattenuated correlation coefficients were 0.35 for men and 0.22 for women in the first group and 0.23 for men and 0.16 for women in the second.^6^ These findings suggest that the difference in reference intakes is less likely to explain the difference in the validity of protein intake. However, dietary intake over 4 consecutive days might not reflect seasonal variation in food intake. In addition, dietary intake might be affected by foods consumed during the preceding day, suggesting a higher probability that a food eaten on a previous day food will be selected again. These limitations were considered when 4-day weighed DRs were used as a reference method.

Several possible explanations for the observed lack of positive correlations between the dietary intake of amino acids and plasma levels can be considered. First, misclassification due to inaccurate measurement may have attenuated the true association. However, since the reproducibility of assays for plasma levels of most amino acids was relatively high, at less than 6% for all intra- and inter-assay CVs except for cystine, aspartic acid, and glutamic acid, the absence of positive correlations was unlikely to be due to measurement error. In the present study, blood samples were left on ice and centrifuged within 30 minutes after collection to minimize the metabolizing effect of metabolic enzymes from blood cells on nonessential amino acids. Nevertheless, we cannot deny the possibility that metabolism of nonessential amino acids might have affected plasma levels. Measurement errors related to the 4-day weighed DRs and the FFQ might also have influenced the observed correlations. However, given that we observed moderate correlation coefficients for amino acid intake from the DR and FFQ, these might not be a major reason for the absence of positive correlations. In addition, supplement use may be an important source of amino acid intake, but intake from supplements was not included in dietary intake in the present study because a database for dietary supplements was not available. Since only one subject took amino acid supplements in the present study, however, supplement use was unlikely to account for misclassification of dietary intake.

Second, several studies have shown that plasma levels of amino acids increase rapidly after eating and then decrease rapidly (eg, within 5 hours) to pre-eating levels (ie, basal levels).^15^^,^^16^ For example, in a study of Japanese women aged 20–25 years using isocaloric test meals, plasma levels of amino acids were increased at 1 and 3 hours after a high-carbohydrate or high-fat meal, but plasma levels at 5 hours after a high-carbohydrate or high-fat meal were almost identical to those before eating, although they continued to increase at 5 hours after a high-protein meal.^15^ In the present study, no positive correlation was found between dietary intake of amino acids and plasma levels, even after excluding subjects with non-fasting samples (less than 8 hours). Although the immediate postprandial effect of dietary protein on plasma amino acid levels has been suggested, our findings indicate that dietary intake of essential amino acids is not a major determinant of basal levels in plasma.

In conclusion, we confirmed that this FFQ has moderate validity in estimating amino acid intake when 4-day weighed DRs were used as a reference standard, suggesting that this FFQ is suitable for ranking individuals living in urban areas in Japan by intake of amino acids. In contrast, the lack of a positive correlation between dietary intake and plasma levels of amino acids suggests that dietary intakes are not major determinants of plasma levels and that plasma levels do not reflect levels of usual dietary intake.

## ONLINE ONLY MATERIAL

Abstract in Japanese.
