# Supplementary material for: Validity of a Self-Administered Food-Frequency Questionnaire for Assessing Amino Acid Intake in Japan: Comparison With Intake From 4-Day Weighed Dietary Records and Plasma Levels
Source: J Epidemiol. 2016 Jan 5;26(1):36–44. doi: 10.2188/jea.JE20150044 (PMC4690739; doi:10.2188/jea.JE20150044)
Supplement: Abstract in Japanese. [file je-26-036-s001.pdf]

日本におけるアミノ酸摂取量評価のための自記式食物摂取頻度調査票の妥当性：4 日間の食事記録調査と血漿濃度との比較

岩崎　基[1]、石原淳子[2,4]、高地リベカ[2,5]、等々力英美[6]、山本浩史[7]、宮野　博[7]、山地太樹[1]、津金昌一郎[3]

[1]国立がん研究センターがん予防・検診研究センター疫学研究部
[2]国立がん研究センターがん予防・検診研究センター予防研究部
[3]国立がん研究センターがん予防・検診研究センター
[4]相模女子大学栄養科学部
[5]新潟大学大学院医歯学総合研究科地域予防医学講座環境予防医学分野
[6]琉球大学医学部医学科衛生学・公衆衛生学分野
[7]味の素株式会社イノベーション研究所

【背景】
アミノ酸の生理学的役割や健康アウトカムへの影響に対する関心は大きく、また高まっている。そこで、疫学研究に適用可能な習慣的なアミノ酸摂取の評価と、習慣的な摂取量と血中濃度との関連を明らかにすることが求められている。今回は、食物摂取頻度調査票から推定した食事由来のアミノ酸摂取量により個人をランキングすることの妥当性を、食事記録調査による摂取量および血中濃度と比較することにより検討した。

【方法】
対象者は、国立がん研究センターがん予防・検診研究センターのがん検診受診者のうち、4 日間の食事記録調査、半定量食物摂取頻度調査票、血漿検体を提供した 139 人である。血漿中アミノ酸濃度は、UF-amino Station system を用いて分析した。

【結果】
食事記録調査と食物摂取頻度調査票から得られたエネルギー調整済みアミノ酸摂取量のスピアマンの順位相関係数は、男性が 0.40 から 0.65、女性が 0.35 から 0.46 であった。食事記録調査のエネルギー調整済み摂取量と血漿中濃度の相関係数は、男性が-0.40 から 0.25、女性が-0.16 から 0.11 であった。同様に男女とも、食物摂取頻度調査票による摂取量と血漿中濃度の間にも正の相関は観察されなかった。

【結論】
今回の食物摂取頻度調査票は、アミノ酸摂取量の推定において、4 日間の食事記録調査を比較基準とした場合に中程度の妥当性があることを確認した。これは、日本の都市部在住者をアミノ酸摂取量によってランキングすることに適していることを示唆している。

キーワード：アミノ酸、食物摂取頻度調査票、血漿濃度、妥当性
